# Restoration of cortical symmetry and binaural function: Cortical auditory evoked responses in adult cochlear implant users with single sided deafness

**DOI:** 10.1371/journal.pone.0227371

**Published:** 2020-01-14

**Authors:** Andre Wedekind, Gunesh Rajan, Bram Van Dun, Dayse Távora-Vieira

**Affiliations:** 1 Otolaryngology, Head and Neck Surgery, School of Surgery, University of Western Australia, Perth, Australia; 2 Fiona Stanley Hospital, Perth, Australia; 3 Department of Otolaryngology, Head & Neck Surgery, Luzerner Kantonsspital, Luzern, Switzerland; 4 The National Acoustic Laboratories, Sydney, Australia; University of Melbourne, AUSTRALIA

## Abstract

**Background:**

Cochlear implantation for single-sided deafness (SSD) is the only treatment option with the potential to restore binaural hearing cues. Significant binaural benefit has been measured in adults by speech in noise and localisation tests, who receive a cochlear implant for SSD, however, little is known on the cortical changes that help provide this benefit. In the present study, detection of sound in the auditory cortex, speech testing and localisation was used to investigate the ability of a cochlear implant (CI) to restore auditory cortical latencies and improve binaural benefit in the adult SSD population.

**Methods:**

Twenty-nine adults with acquired single-sided deafness who received a CI in adulthood were studied. Speech perception in noise was tested using the Bamford-Kowal-Bench speech-in-noise test, localisation ability was measured using the auditory speech sounds evaluation (AδE) localisation test and cortical auditory evoked responses, comparing N1-P2 latencies recorded from the normal hearing ear and cochlear implant were used to investigate the synchrony of the cortical pathway from the CI and normal hearing ear (NHe) with binaural hearing function.

**Results:**

There was a significant improvement in speech perception in noise in all spatial configurations S0/N0 (Z = -3.066, p<0.002), S0/NHE (Z = -4.031, p<0.001), SCI/NHE (Z = -3.851, p<0.001). Localization significantly improved when tested with the cochlear implant on (p<0.001) with a shorter duration of deafness correlating to a greater improvement in localisation ability F(1:18) = 6.854; p = 0.017). There was no significant difference in N1-P2 latency recorded from the normal hearing ear and the CI.

**Conclusion:**

Cortical auditory evoked response latencies recorded from the CI and NHe showed no significant difference, indicating that the detection of sound in the auditory cortex occurred simultaneously, providing the cortex with auditory information for binaural hearing.

## Introduction

Single-sided deafness (SSD) is a condition where an individual has a severe-profound sensorineural hearing loss in one ear and normal hearing on the contralateral side. This deprives the individual of access to binaural cues: binaural squelch, binaural summation and the head-shadow effect [1]. The real-world impact of these deficits is poor speech understanding in noise and impaired localisation. Cochlear implantation in SSD started as a treatment for adults with intractable tinnitus [2–5]. However, hearing improvement was also obtained by these patients, opening the door for several research groups to further investigate the restoration of binaural hearing benefits including sound localisation, speech perception in noise and self-perceived hearing abilities [6–11]. Early study by Vermeire and Van De Heyning (2009) showed that CI could improve hearing in SSD patients. Arndt et al (2011) compared CI to other modalities of hearing rehabilitation in SSD patients with less than 10 years of duration of deafness. They concluded that CI for SSD provided superior outcomes compared with other treatment options (bone-conduction implants or contralateral-routing of signal hearing aids) and that the signal from the CI does not interfere with the normal hearing ear’s ability to understand speech [8]. Távora-Vieira et al. (2015) demonstrated that SSD CI users have a significant improvement in localization abilities with CI on compared to CI off and duration of deafness had no significant effect on the outcomes. More recently, Prejban et al (2018) found that there was no significant difference in lateralization ability in SSD CI users compared with a normal-hearing group. Further, an improvement of 1.6dB was seen in speech reception thresholds (SRT) in CI recipients when wearing their implant compared to not wearing it, with CI experience influencing the measured improvement [12]. A long-term follow up study by Mertens et al. (2016) demonstrated that SSD CI recipients retained benefit binaural effects when tested a minimum of 3 years after implantation[13]. Supporting this is a multi centre study using thirty-four SSD CI recipients who maintained binaural improvement in the long term, with a significant improvement in localisation (in the horizontal plane), speech in noise and subjective hearing performance 4–10 years after implantation [14].

Predicted outcomes of CI for SSD have in the past been extrapolated from the bilaterally deaf population, with results initially thought to be dependent on duration of deafness of the ear to be implanted [15]. Recent research from our group and other implant units challenge this, demonstrating significant improvements in speech-in-noise and localisation abilities even after a long duration of severe-profound hearing loss in one ear (>10years) [16,17]. The difference in candidacy between bilaterally deaf and SSD may be attributable to the bilateral cortical activation by the better hearing ear. Boisvert et al (2012) reviewed the outcomes of ten adults with severe bilateral hearing loss who had worn a hearing aid in only one ear (for a minimum of 15 years). Each individual received sequential bilateral CI’s and underwent standardized speech recognition testing after one year. No significant difference was recorded in speech recognition scores between the two ears [18], indicating that auditory deprivation in one ear did not influence CI outcomes. Support for the preservation of cortical integrity from unilateral hearing has been supported by multiple authors who have demonstrated that CI outcomes are more closely related to the hearing in the better ear rather than the preoperative hearing of the implanted ear[19–21].

Cortical auditory evoked potentials (CAEPs) are a series of negative and positive deflections referred to as the N1-P2 complex with latencies roughly around 100-200ms after stimulus onset [22]. The N1-P2 complex does not require attention from the participant and correlates more closely with behavioural measures than auditory evoked potentials which arise from the auditory nerve or brainstem [23]. Further, it is a surrogate marker of cortical maturation and neural plasticity after implantation [24]. P2 latency is associated with speech perception with poor CI performers demonstrating a delayed P2 latency compared with normal hearing controls [25–27] and may also correlate with the effects of auditory training and experience [28–30]. In post-lingual bilaterally deaf adult CI users who received an implant in one ear, an increase in N1 amplitude and a reduction in N1 latency was recorded over the first 59 weeks with the greatest change occurring in the first 8 weeks post implantation. However, N1 latencies recorded from the CI were still significantly longer then when compared to a normal hearing control group [31].

Binaural hearing benefits which include speech understanding in noise and localization requires cortical representation of both ears. However, as CI for SSD is a new occurrence there is limited research into how the sound delivered through the implant compares to the normal hearing ear at a cortical level. This study was devised to investigate if the SSD CI users are receiving synchronised input at cortical level. Latency of cortical responses obtained from the electrical stimulations will be compared to those obtained from the normal hearing ear. It is hypothesised that better localization ability and better speech understanding in noise will correlate positively to more synchronised cortical activation.

## Method

### Subjects

Twenty-nine adults (14 men and 15 women) with acquired post-lingual SSD and CI users, with a CI experience of at least 3 months, were requited for this study. Mean age at testing was 59.8 years ± 14.2 (range 27–81 years). Mean duration of deafness was 8.9 years (range 0.2–41 years) and CI experience 3.2 years (range 0.5–8.7 years).

The cause of deafness was; idiopathic sudden sensorineural hearing loss (n = 16), Meniere’s disease (n = 4), mumps (n = 2), head trauma (n = 1), fistula (n = 1), superior semi-circular canal dehiscence surgery (n = 1), meningitis (n = 1) and unknown (n = 3) (see Table 1 for demographics).

**Table 1:** Demographic data.

ID	Age (years)	Duration of deafness (years)	Cochlear implant experience (years)	Sex	Ear	Aetiology	Non-implanted ear	Implanted ear
1	66	2	5.5	M	L	head trauma	31	90
2	65	40	5.5	F	L	ISSNHL	14	80
3	80	0.3	5.4	M	R	ISSNHL	36	84
4	65	20	5.4	M	L	MD	24	NR
5	70	1	5.1	M	L	ISSNHL	21	97
6	63	0.4	5.0	M	R	ISSNHL	19	72
7	57	0.9	4.6	F	L	ISSNHL	7	67
8	46	0.3	4.4	F	R	fistula	32	NR
9	49	12	4.2	M	L	ISSNHL	8	72
10	81	1.5	3.6	F	L	ISSNHL	32	NR
11	48	35	3.5	F	R	mumps	11	NR
12	63	1	3.5	M	L	MD	41	76
13	32	17	3.4	F	R	ISSNHL	26	NR
14	50	0.6	2.8	M	L	unknown	16	83
15	41	0.8	1.8	F	R	ISSNHL	8	NR
16	64	1.5	1.8	M	R	ISSNHL	19	83
17	60	41	8.7	F	L	mumps	16	NR
18	76	3	1.6	F	L	ISSNHL	13	79
19	74	1	1.1	M	R	ISSNHL	33	85
20	52	5	2.2	M	R	ISSNHL	31	NR
21	55	30	1.2	M	L	ISSNHL	11	NR
22	75	0.8	1.0	M	R	ISSNHL	9	63
23	68	0.6	4.3	F	L	MD	26	73
24	70	2.5	1.1	F	R	ISSNHL	27	84
25	48	0.2	1.2	F	R	Meningitis	39	64
26	79	1.4	0.7	M	R	SSCD surgery	33	77
27	27	21	0.8	M	R	congenital partial deafness	35	83
28	50	5	0.5	M	R	MD	14	NR
29	41	2	0.6	F	L	unknown	10	NR

ISSNHL: Idiopathic Sudden Sensorineural Hearing Loss, MD: Meniere’s Disease, SSCD: Superior semi-circular canal dehiscence.

They all were implanted with the MED-EL Standard, FLEXsoft or FLEX [28] electrode array and received an OPUS 2, RONDO or SONNET speech processor (MED-EL, Innsbruck, Austria). They use their speech processors on a full-time basis.

Ethics approval was obtained from the South Metropolitan Health Ethics Committee (reference number: 335) Subjects have given their written informed consent to participate in this study.

### Cortical auditory evoked potentials (CAEPs)

CAEPs were recorded using HEARlab™ System (Frye Electronics) with the active electrode at the vertex (Cz), referenced to the mastoid and the forehead set as the ground. Electrode impedance was kept below 5kohms.

Four speech tokens /m/, /g/, /t/ and /s/ were presented at 55dB SPL in free-field with the participants seated 1 metre 0° azimuth in front of a loud speaker. The speech signal duration was 30ms for /m/, 21ms for /g/, 30ms for /t/ and 50ms for /t/. Inter stimulus interval was 1125ms, and the minimum number of acceptable epochs for responses to each speech signal was set at 200. Testing for each speech token was terminated automatically when 200 accepted epochs were reached, or manually when the p-value was ≤ 0.05. The time window for the recording was from 200ms before the triggering stimulus (baseline) to 600ms after the stimulus onset. The residual noise level was kept below 3.2 μV. Baseline correction was applied to each individual epoch based on the average over 100ms prior to stimulus onset. HEARLab applies an automatic statistical criterion (Hotelling’s T statistical test) to determine the presence or absence of CAEPs [32].

N1 and P2 peak latency was calculated. The maximum/minimum values were selected from the time windows of interest (50-150ms post-stimulus for N1 and 150-250ms post-stimulus onset for P2) [33].

Results were recorded from four different electrode montages:

1. CI on, contralateral pathway–reference electrode placed on the mastoid contralateral to the CI side;
2. CI on, ipsilateral pathway–reference electrode placed on the mastoid ipsilateral to the CI;
3. CI off, contralateral pathway–reference electrode placed on the mastoid contralateral to the normal hearing ear; and
4. CI off, ipsilateral pathway–reference electrode placed on the mastoid ipsilateral to the normal hearing ear.

The CI pathway was tested with the normal hearing ear masked at 70dB HL constant broadband noise presented through an insert earphone. This level of masking has previously been shown to be an effective masker of CAEP [34]. CI off pathway was tested with the CI removed.

If a N1-P2 complex was not recorded and reprogramming of the CI was needed to provide the participant access to certain speech sound, CAEP potential were reviewed at a maximum of 28 days between after CI programming [34].

### Speech perception in noise testing

A 3-speaker setup in free field was used with speakers placed 1 metre away at 0°, 90° and -90° to evaluate the hearing performance in noise. The speech material was the Bamford-Kowal-Bench adaptive speech-in-noise test (BKB-SIN) [35] which investigates the speech-in-noise ratio needed to repeat 50% of words correctly. Speech was presented at 65dB SPL. Three spatial configurations were used for testing; S_0_/N_0_, speech and noise presented from the front; S_0_/N_HE_, speech presented from the front and noise presented on the normal hearing ear side; and S_CI_/N_HE_, speech presented to the CI and noise presented on the normal hearing side. Speech-in-noise testing compared the CI on condition with the CI off condition.

### Localisation

Localisation was measured using the auditory speech sounds evaluation (AδE) localisation test (PJ Govaerts, Antwerp, Belgium). In a soundproof booth, a 4000Hz narrowband sound was simultaneously presented through two speakers placed at 60 and -60 degrees from the subject. The presentation level through each speaker randomly differs creating an interaural level difference (ILD), allowing the illusion of sound to be somewhere on the azimuth between the two speakers. This allowed 13 localisation points (2 true speakers and 11 sham speakers) placed at 10-degree intervals [36,37]. The subjects would report which speaker they thought the sound was coming from, with each response being entered into computer software which calculated the median values and root mean square (RMS), as a measure of a subject’s localisation ability. A lower RMS error represents better localisation skills. AδE was used in this study as it relies on ILDs, which is thought to be the binaural cue people with CI’s primarily rely on for localisation [38].

### Statistical methods

The non-parametric Wilcoxon signed-rank test was used to test for a significant difference between CI on and CI off conditions to examine improvements in (1) localization, (2) speech understanding in noise and (3) CAEPs measurement. The data distribution was checked using the Kolmogorov-Smirnov test. Missing data were treated as missing values. Because of 4 pairwise comparisons per CAEPs group, the Bonferroni correction method must be used when interpreting the achieved p-values. Hence a p-value ≤0.0125 instead of ≤0.05 is considered as significant.

IBM SPSS statistics (IBM, Armonk, New York) was used for the analyses and graphs were created in Microsoft Office Excel 2010 (www.microsoft.com).

## Results

### Speech in noise

**Fig 1:**
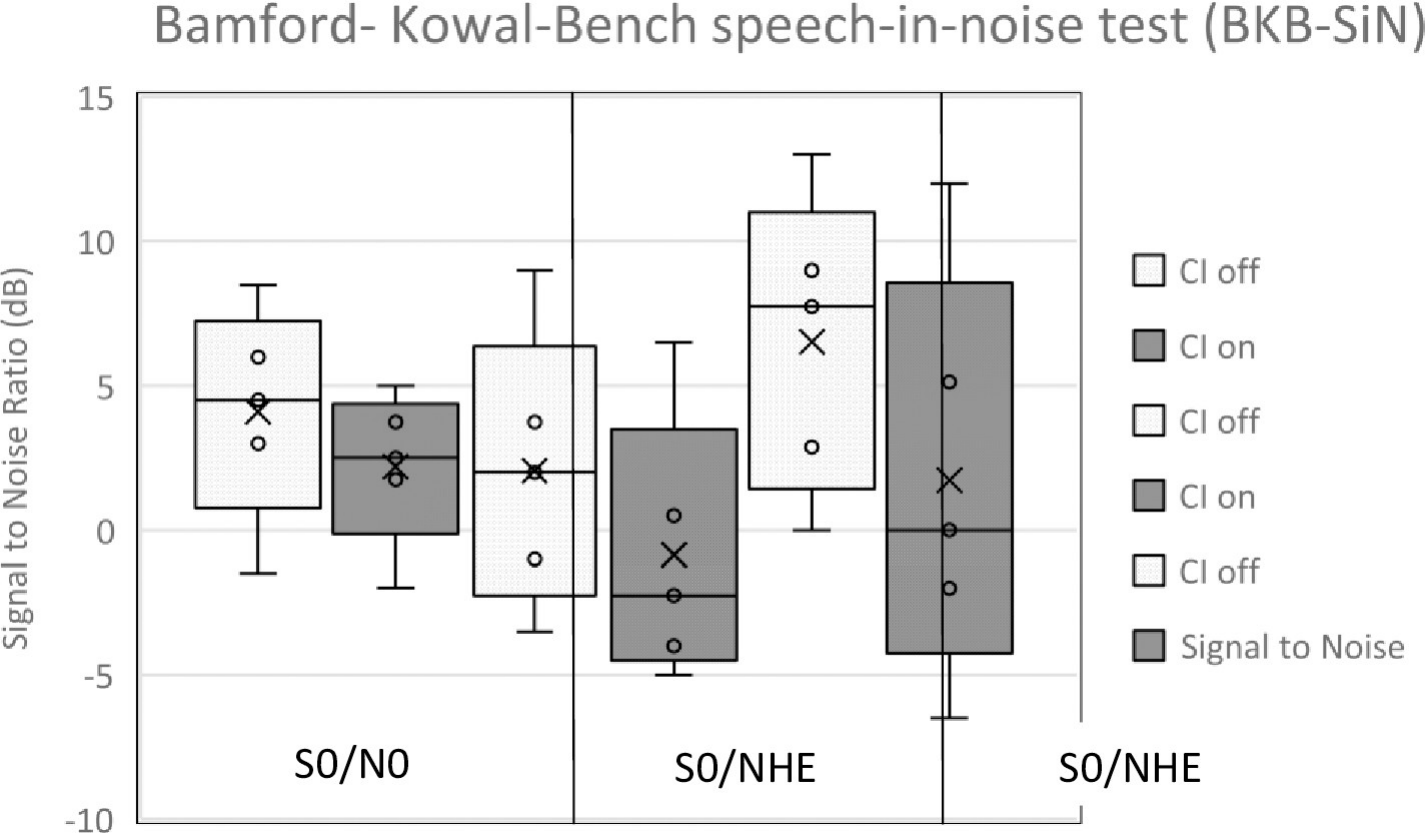
Bamford-Kowol-Bench speech-in-noise test results for CI on and CI off conditions from 3 different spatial configurations (S0/N0, S0/NHE and SCI/NHE).

A Wilcoxon signed rank test indicated that a significant improvement was seen in all three spatial configurations while using the cochlear implant: S_0_/N_0_ (Z = -3.066, p<0.002), S0/N_HE_ (Z = -4.031, p<0.001), S_CI_/N_HE_ (Z = -3.851, p<0.001) see Fig 1. The results of a Multivariate ANOVA indicate that the amount of time someone has a cochlear implant (duration of cochlear implant use) has a significant effect on speech in noise for the S_0_/N_0_ (F(1:18) = 6.137; p = 0.023) but no significant difference was seen for S0/N_HE and_ S_CI_/N_HE_ conditions _(_S0/N_HE_ (F(1:18) = 1.038; p = 0.322, S_CI_/N_HE_ (F(1:18) = 0.140; p = 0.712. Duration of deafness prior to implantation did not significantly influence any of the speech in noise configuration results (S_0_/N_0_ (F(1:18) = 0.044; p = 0.837, S0/N_HE_ (F(1:18) = 0.704; p = 0.0.412, S_CI_/N_HE_ (F(1:18) = 0.351; p = 0.561.

### Localisation

**Fig 2:**
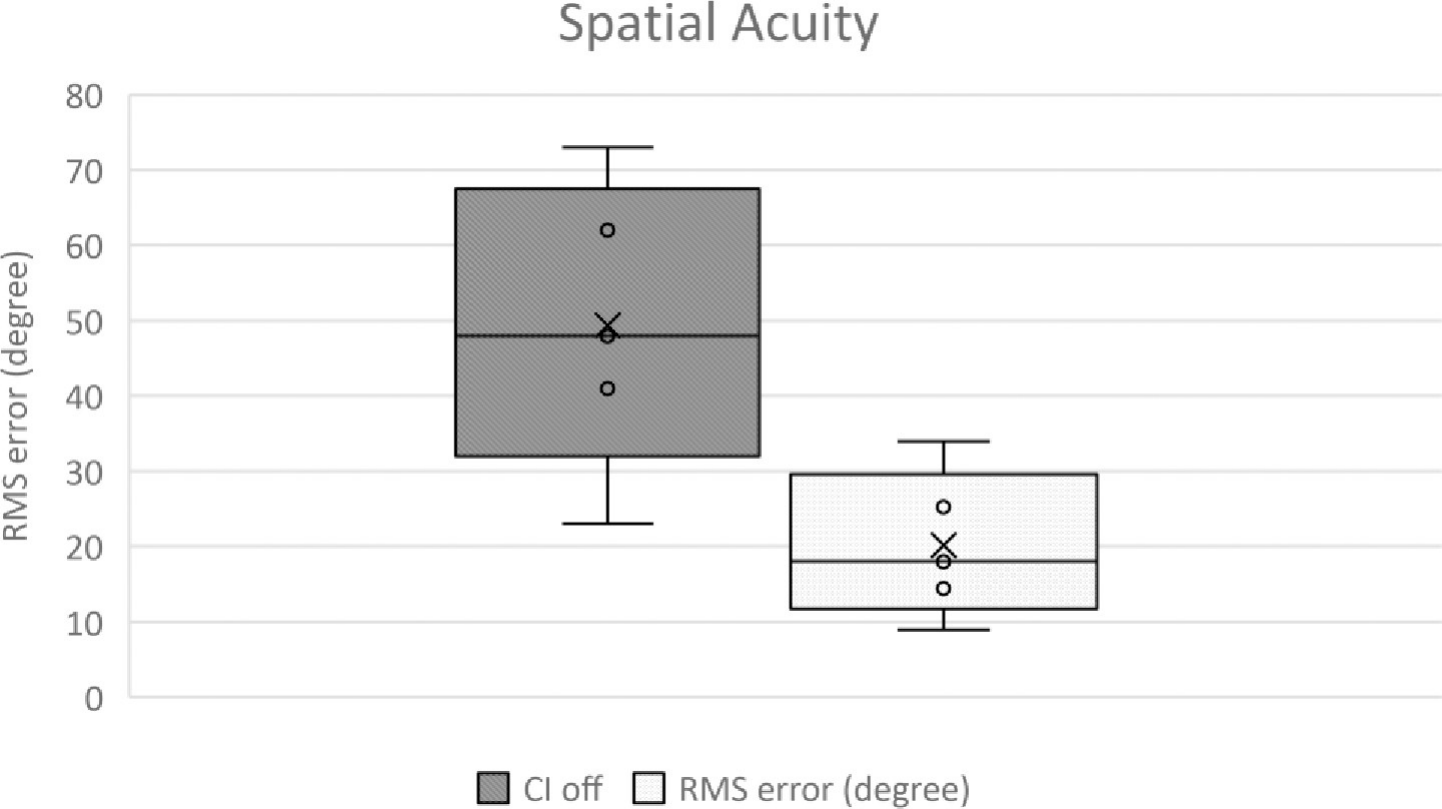
Mean root mean-square (RMS) error with CI on and CI off. Lower RMS represents better localisation skills.

A Wilcoxon signed-rank test determined that significant improvement was achieved when wearing the cochlear implant (p<0.001) as shown in Fig 2. Duration of deafness prior to implantation was correlated with localisation ability (F(1:18) = 6.854; p = 0.017), however, the amount of time the participants had had their implant was not associated with localisation results (F(1:18) = 0.026; p = 0.874).

### Cortical responses

#### Baseline measure

**Fig 3:**
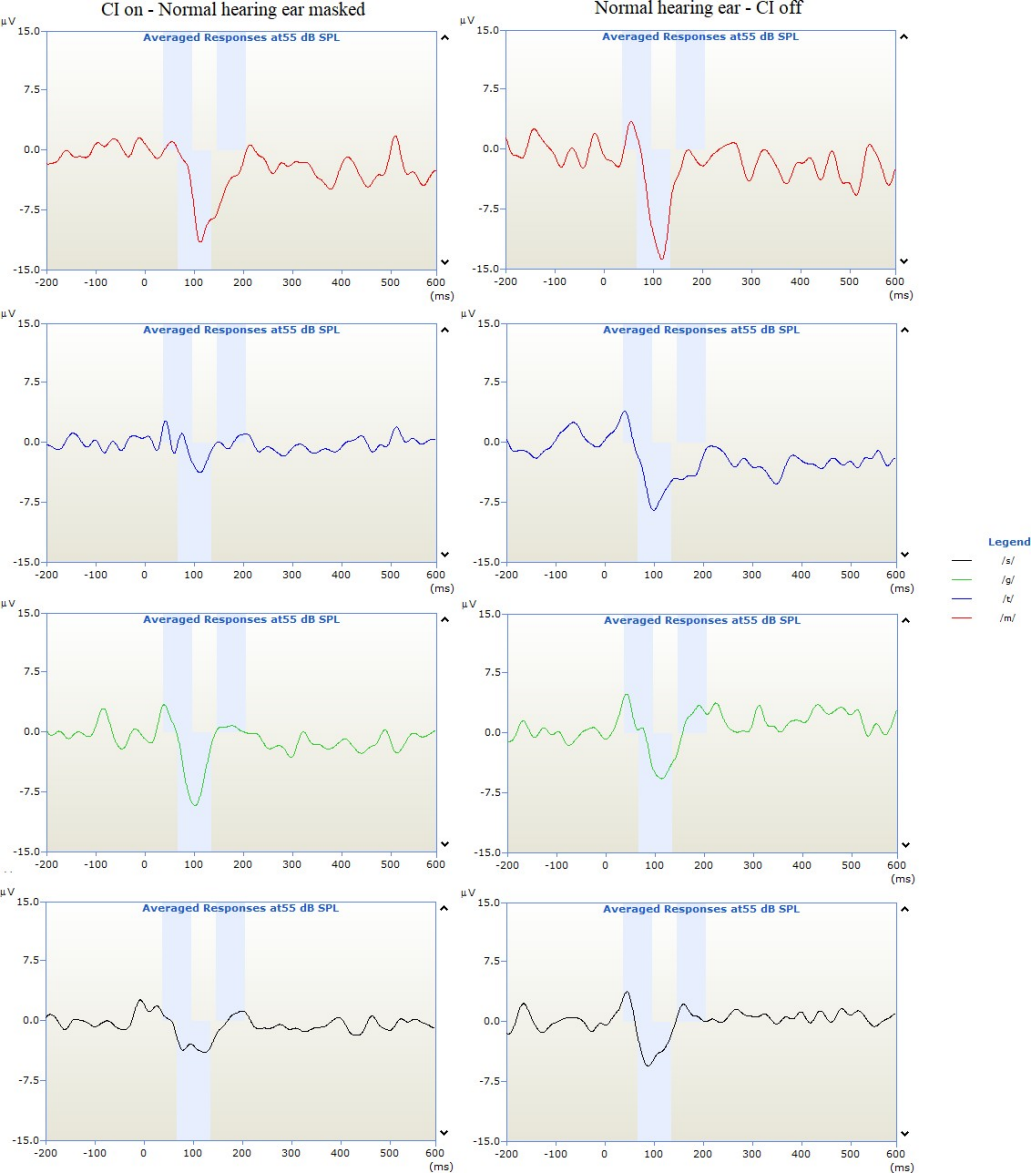
Example of CAEP traces for speech tokens /m/, /t/, /g/ and /s/ with CI off and normal hearing ear masked at 70dB HL broadband noise.

CAEP measurement from the CI was obtained with the normal hearing ear masked at 70dB HL. Fig 3 shows an example of the recording in which the CI is off and the normal hearing ear is masked with the N1-P2 complex being absent.

#### Quality of recording

**Fig 4:**
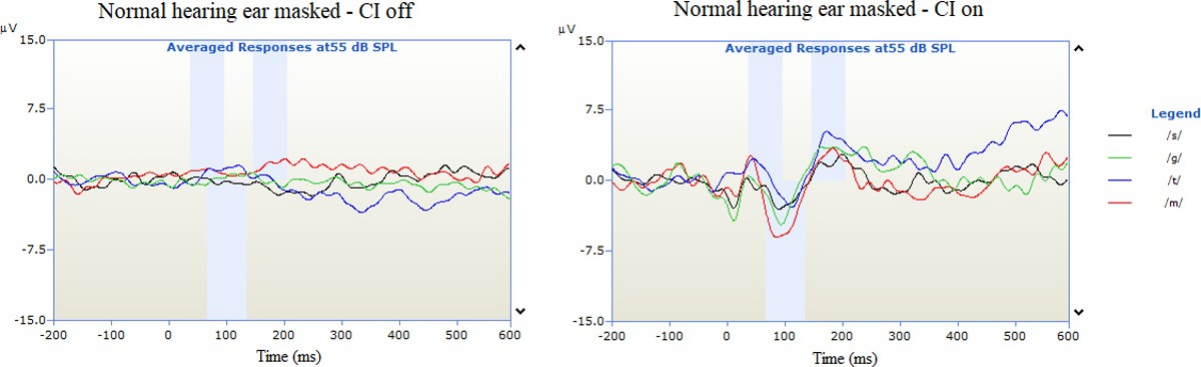
Example of CAEP traces from the normal hearing ear and CI.

Artefact interference did not influence the quality of the recordings. Fig 4 shows an example of CAEP recordings taken with the CI on and CI off.

#### N1 and P2 latencies

**Fig 5:**
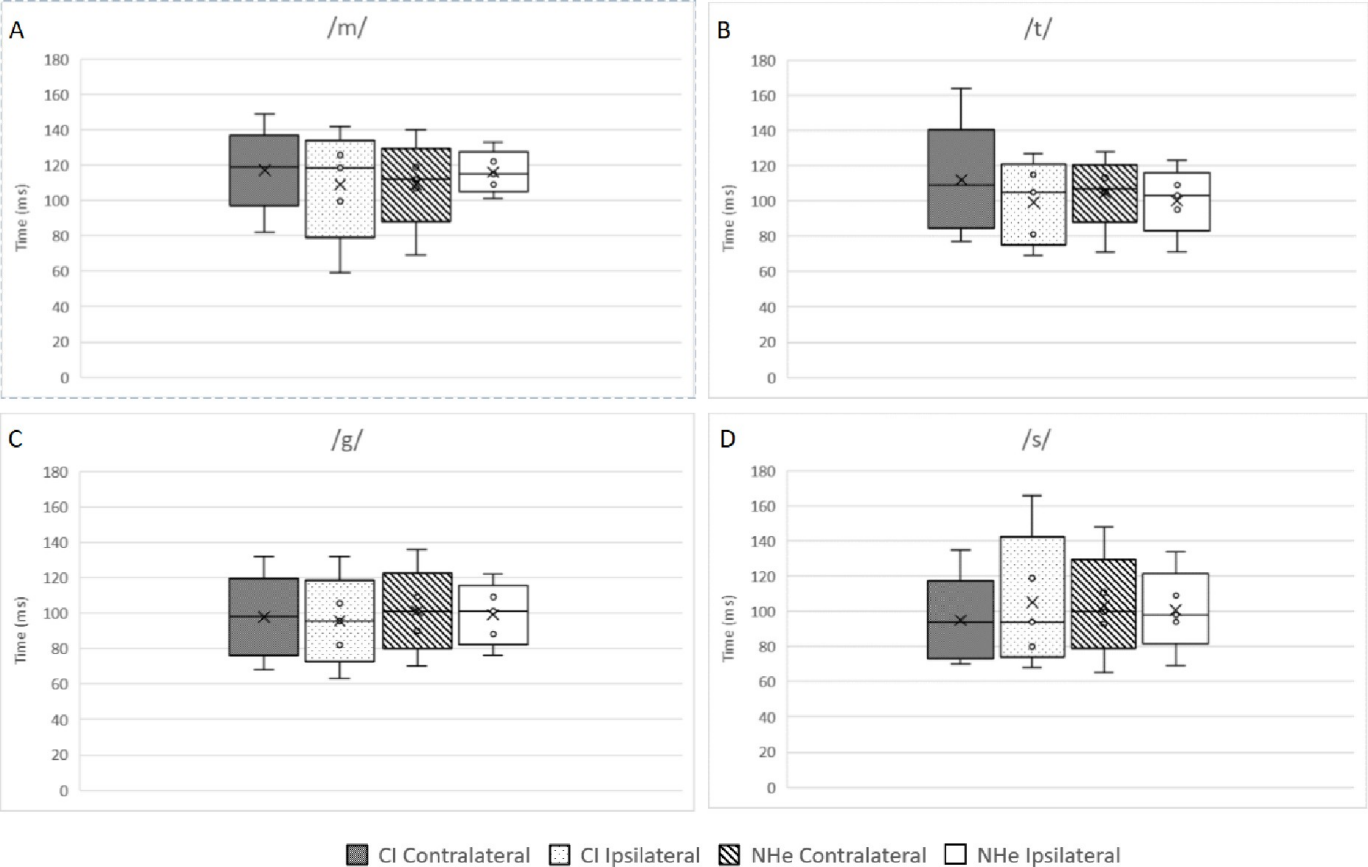
Latencies (ms) recorded for N1 from four different electrode montages for each speech token. Median values are the horizontal line, mean value as the black x.

**Fig 6:**
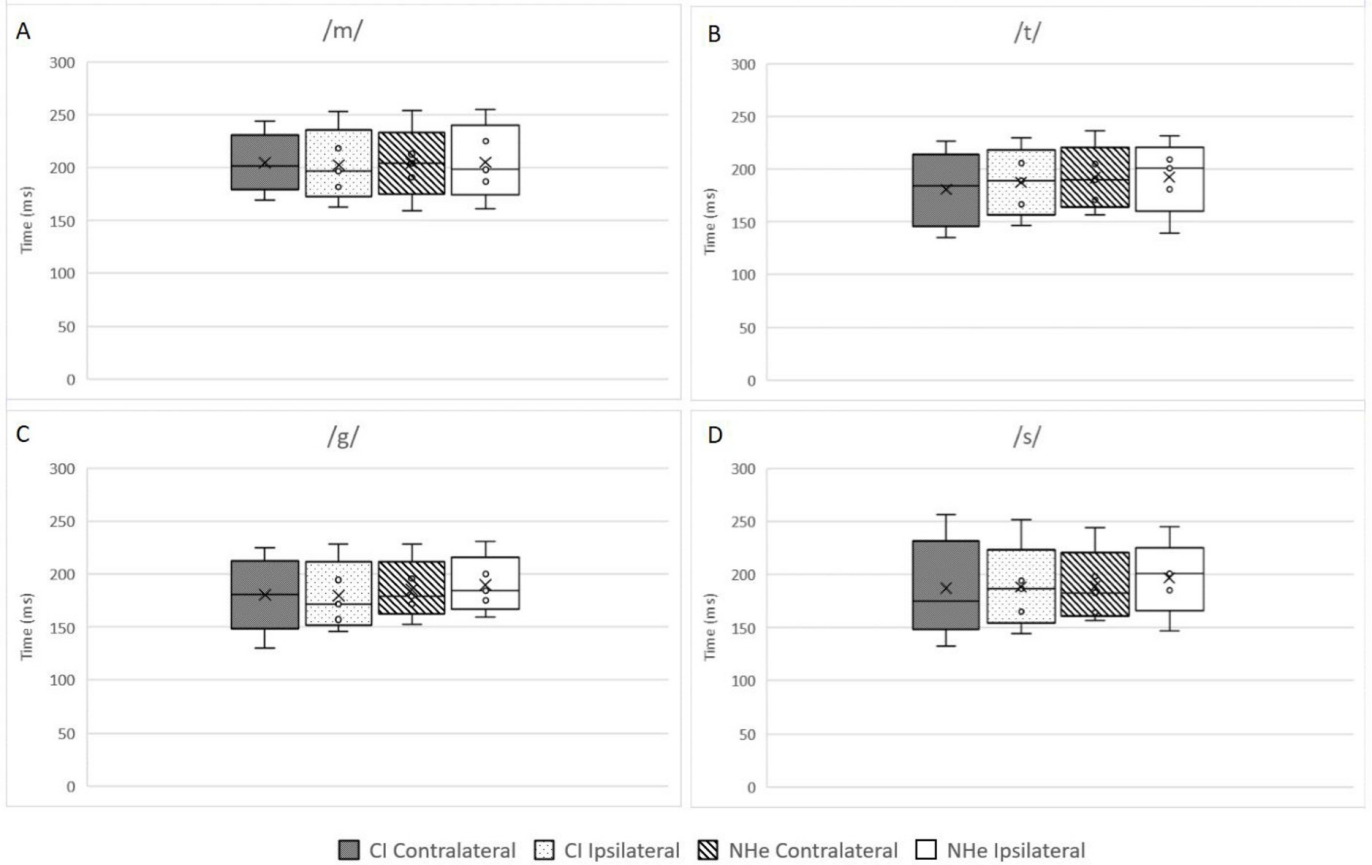
Latencies (ms) recorded for P2 from four different electrode montages for each speech token. Median values are the horizontal line, mean value as the black x.

Group average for N1 and P2 latencies were calculated for each speech token. Speech token /m/ consistently had a longer N1and P2 latency compared to /g/, /t/, and /s/ (shown in Figs 5 and 6). N1-P2 latencies obtained from the CI were compared with the corresponding electrode montage from the normal hearing ear (CI ipsilateral pathway compared to Normal hearing ear ipsilateral pathway and CI contralateral pathway compared to Normal hearing ear contralateral pathway).

Participants were split into three groups; <2years CI experience, 2-5years CI experience and >5years CI experience. Using a Wilcoxon signed-rank test, significant difference years CI experience was seen when comparing P2 ipsilateral pathway for speech token /m/ (p = 0.042) for the group who had >5years and when comparing speech token /t/ for P2 contralateral pathway as shown in Table 2. Fig 5 illustrates similar distribution patterns between each electrode montage for all participants for each of the speech tokens. Speech tokens /g/ and /t/ produced stable results. Speech token /m/ elicited greater variability in recordings from the CI while less recordings were elicited by speech token /s/ from both the CI and normal hearing ear.

**Table 2:** Wilcoxon signed-rank test showing the difference between CI-on and CI-off.

CI experience		N1_Contra with CI-off—N1_Contra with CI-on	P2_Contra with CI-off—P2_Contrawith CI-on	N1_Ipsi with CI-off—N1_Ipsi with CI-on	P2_Ipsi with CI-off—P2_Ipsi with CI-on
**/m/**					
< 2 yrs	Z	-1.379	-1.245	-.866	-.801
	p-values (2-sided)	.168	.213	.386	.423
2 to 5 yrs	Z	-.510	-1.682	-1.377	-1.362
	p-values (2-sided)	.610	.093	.169	.173
> 5 yrs	Z	-.169	-.423	-.135	-2.032
	p-values (2-sided)	.866	.672	.893	.042*
**/t/**					
< 2 yrs	Z	-.979	-.802	-.578	-.757
	p-values (2-sided)	.327	.423	.563	.449
2 to 5 yrs	Z	-1.380	-2.245	-1.472	-.314
	p-values (2-sided)	.168	.025*	.141	.753
> 5 yrs	Z	-.841	-.943	-.365	-1.826
	p-values (2-sided)	.400	.345	.715	.068
**/g/**					
< 2 yrs	Z	-1.201	-.051	-.622	-1.247
	p-values (2-sided)	.230	.959	.534	.212
2 to 5 yrs	Z	-.766	.000	-1.173	-1.376
	p-values (2-sided)	.444	1.000	.241	.169
> 5 yrs	Z	-.676	-.507	-1.214	-1.214
	p-values (2-sided)	.499	.612	.225	.225
**/s/**					
< 2 yrs	Z	-.254	-.338	-1.185	-1.355
	p-values (2-sided)	.799	.735	.236	.176
2 to 5 yrs	Z	-.135	-.813	-.135	.000
	p-values (2-sided)	.893	.416	.893	1.000
> 5 yrs	Z	-1.826	-1.604	-1.604	-1.604
	p-values (2-sided)	.068	.109	.109	.109

*significant difference between CI-on and CI-off.

The difference in latency for N1 and P2 between the CI and normal hearing ear (for both the ipsilateral and contralateral pathways) for each speech token was calculated. A Pearson’s correlation found no relationship between latency difference and localisation improvement (ipsilateral pathway p = 0.091 to p = 0.992; contralateral pathway p = 0.096 to p = 0.909). A larger difference in N1 latency recorded from speech token /m/, when comparing the contralateral pathways, correlated with an increased SiN improvement for S0/N_HE_ (p = 0.002) and S_CI_/N_HE_ (p = 0.036). Increased N1 latency difference recorded from speech token /s/ ipsilateral pathways correlated with SiN improvement for the S0/N_HE_ (p = 0.033) paradigm. No other significant correlation was found.

## Discussion

Cochlear implants have been used to rehabilitate severe to profound sensorineural hearing loss for over 30 years. With improvements in technology and surgical techniques the outcomes have improved over time and the candidacy criteria expanded substantially over the years. As CI candidacy evolves we are seeing impressive functional outcomes from the SSD population where the brain integrates the electrical stimulation from one auditory pathway with the normal acoustic stimulation from the normal hearing ear to achieve binaural hearing benefit. These benefits include better speech understanding in noise and localisation.

CAEPs have been widely used to evaluate the benefit of cochlear implants in young children where speech perception testing cannot be performed. In our centre, CAEPs have been introduced as a tool to evaluate optimal fitting of CI in SSD users [34]. It is expected that if we provide speech detection at a cortical level we may facilitate the progress post-CI in terms of speech understanding. However, the question this study aims to answer is if there is a difference between the normal hearing ear and the electrical stimulation for speech detection at a cortical level.

Localisation relies on binaural cues (interaural level difference–ILD and time differences–ITD), with auditory neurons becoming sensitive to binaural cues in early development [39]. ILD is dependent on the amount of excitation received from each ear, therefore the degree of asymmetrical hearing reduces the sensitivity of localisation [40]. In this study, a significant (p<0.001) improvement in localization ability was found in the CI-on scenario compared to the CI-off condition suggesting the restoration of binaural ILDs. Of note is that duration of deafness before cochlear implantation was associated with the degree of improvement, with a shorter duration of deafness before implantation significantly correlating to improved localisation ability. However, within our demographic, our long duration of deafness participants also lost their hearing early in life making it difficult to separate whether the primary factor influencing localisation ability is the duration of deafness or the age of onset of SSD. We also found that cochlear implant experience did not correlate with localisation ability. This supports findings from Grantham et al (2007) who found no change in localisation ability in bilateral implantees who had their cochlear implant for 5 months compared to being tested at 15 months [38].

Binaural input improves speech understanding in noise by binaural squelch and summation. The squelch effect is the ability of the brain to selectively filter noise from the desired sound, more so when noise and speech are in different azimuthal or vertical locations due to differing inter-aural phase and time differences [41], for CI users these timing and phase cues are largely unavailable. In normal-hearing individuals the advantage of binaural squelch for speech in noise is a 2-5dB gain in signal to noise ratio (SNR) [42]. Binaural summation is a phenomenon where sound is perceived louder when listening with two ears compared to listening with one. People with SSD are disadvantaged when sound is projected directly to their non-hearing ear, due to the physical barrier of their head. This is called the head shadow effect and can attenuate sound by 10-16dB [1]. We found a significant improvement over all speech in noise configurations S_0_/N_0_ (p = 0.002), S_0_/N_HE_ (p<0.001), and S_CI_/N_HE_ (p<0.001) with an improvement in SNR of 1.76 ± 2.4 for S_0_/N_0_, 3.02 ± 2.6 for S_0_/N_HE_ and 4.89 ± 5.0 S_CI_/N_HE_. These results indicate that the electrical signal received from the implanted ear integrates with the opposite acoustic sound in the normal hearing ear to provide binaural hearing benefit. Our findings are in line with published literature, Friedman et al (2016) using a cohort of 9 adults with acquired SSD found an improvement in SNR of 2dB ±0.8 for S_0_/N_0_ and 4.6dB ± for S_0_/N_HE_ speech in noise configurations after CI [43]. Rahne et al (2016) found an improvement in speech reception threshold in noise as early as 3 months after implantation [44].

Multivariate analysis demonstrated that the amount of improvement seen in the S_0_/N_0_ condition is correlated to the amount of time that the participant had their implant (F(1;18) = 6.137; p = 0.023). S_0_/N_0_ speech in noise testing relies on binaural summation to provide an advantage, which with time and reestablishment of the neural pathway through the CI stimulation provides this summation benefit. Litovsky et al (2018) found no significant summation effect after implantation in their cohort of 6 SSD adult participants when using the AzBIO sentence test with a SNR of 0dB [45]. Our results differ from this, showing a summation effect present as early as 3 months post implantation, with larger improvements seen in participants who have a longer duration of CI experience. One explanation for such difference is that in our study, by using the adaptive BKB-SiN speech test tracking the 50% correct point on the psychometric function, we were able to measure smaller improvements using a more sensitive testing modality.

The presence of CAEPs to speech stimuli provides an objective indication of the subject’s ability to access speech sounds. By using the speech tokens /m/, /t/, /g/ and /s/ it ensures that sound information is obtained over the entire speech spectrum. Using a Wilcoxon signed-rank test, cortical auditory evoked latencies from N1 and P2 revealed no significant difference between responses recorded from the cochlear implant and normal hearing ear, as shown in Fig 3. Although this does not provide information on whether the brain processes sound differently (between the electrical stimulation of a CI and acoustic in the normal-hearing ear) it does indicate that sound is received at the primary auditory cortex simultaneously, providing the framework for binaural hearing. Initially, a mismatch was expected due to the difference in how auditory information is provided to the auditory nerve. Previous studies support our results which show a strong correlation between CAEP latencies and speech perception scores [46]. Legris et al (2018) recorded cortical changes from nine SSD individuals who had received a CI, using a loud speaker to present a /ba/ sound simultaneously to the normal hearing ear and CI, this was then compared to a normal hearing cohort. They observed increased bilateral cortical activation which, coupled with an improvement in functional speech in noise testing, may indicate the restoration of binaural cortical function[47]. Cortical responses obtained from the CI in this study further support these findings, coupled with the improvement in speech in noise and localisation. However, our methodology compares CAEPs recorded from the CI to responses from the normal hearing ear making each participant their own control.

When comparing the individual latency differences, we see that the speech tokens /g/ and /t/ produced a large number of significant responses with reduced variability compared to /m/ and /s/ (between CI on and CI off). The stimulus /m/ produced an increased number of variable cases, which is consistent with Kosaner et al (2018) who found that CAEPs produced by /m/ speech tokens were significantly less present and produced longer latencies compared to those recorded from /t/ and /g/ in children who had received a CI for congenital bilateral deafness. [48] Although there was no significant difference in N1 and P2 group average latencies, a reduced number of recordings were available when using the /s/ speech token. This could be due to our inclusion criteria where the four-frequency average (500Hz, 1kHz, 2kHz and 4kHz) of the good ear must be equal to or under 25dBHL. In some cases, there was a high frequency hearing loss preventing the generation of a cortical response when the CI was off. Our initial hypothesis was that individuals with a long duration of deafness before implantation would explain the individual variability in latency (with the individuals with a long duration of deafness contributing to the outliers who had a large latency difference between CI on and CI off) however, no trend was found.

All participants in this study were born with bilateral hearing, which leads to the assumption of established bilateral auditory pathways with normal cortical organisation until the onset of SSD. Unlike congenital bilateral deafness, duration of SSD did not influence N1-P2 latencies. This supports our previous findings, which suggest that cortical reorganisation and reactivation of auditory pathways to the auditory cortex can occur in adulthood even after many years of hearing loss or deafness, indicating that the presence of an established binaural auditory pathway is the main determinant of CI outcomes in the SSD population and not duration of SSD.

### Study limitations

In this study, auditory stimuli were delivered in free field via a loud speaker. Although masking of the normal hearing ear produces no significant cortical auditory evoked activity, we cannot be sure that masking has not added to the neural activation when the CI was on.

The CI users in this study have varied CI experience and therefore we are not able to report on when their was no significant difference between CAEP latencies of the CI and NHe.

No significant difference in P2 latency was found when comparing cortical latencies from the CI to the normal hearing ear. As P2 latency has been shown to correlate with CI speech performance, further research to evaluate the CI alone performance and the correlation or lack of P2 latency in SSD would be beneficial. Due to the scope of this paper [1]individual ear speech testing was not investigated.

## Conclusion

Cortical auditory evoked response latencies recorded from the CI and NHe showed no significant difference, indicating that the detection of sound in the auditory cortex occurred simultaneously, providing the cortex with auditory information for binaural hearing.

## Supporting information

S1 Fig (DOCX)
