# Supplementary figures and images for: Restoration of cortical symmetry and binaural function: Cortical auditory evoked responses in adult cochlear implant users with single sided deafness
Source: PLoS One. 2020 Jan 14;15(1):e0227371. doi: 10.1371/journal.pone.0227371 (PMC6959557; doi:10.1371/journal.pone.0227371)

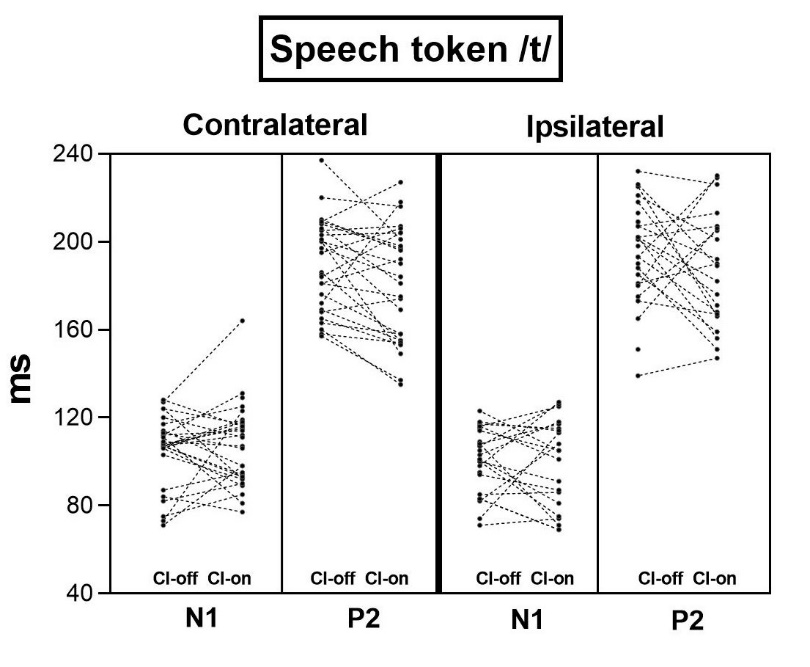

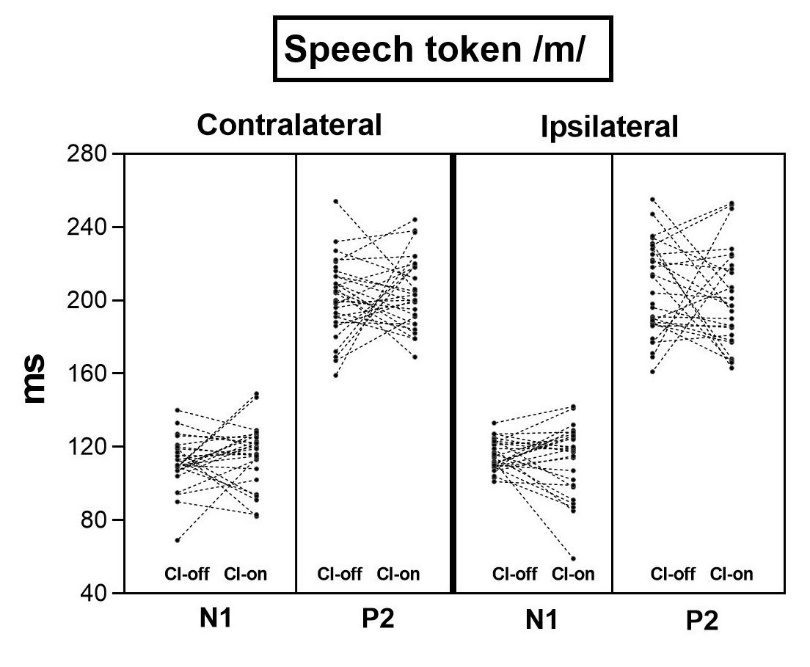


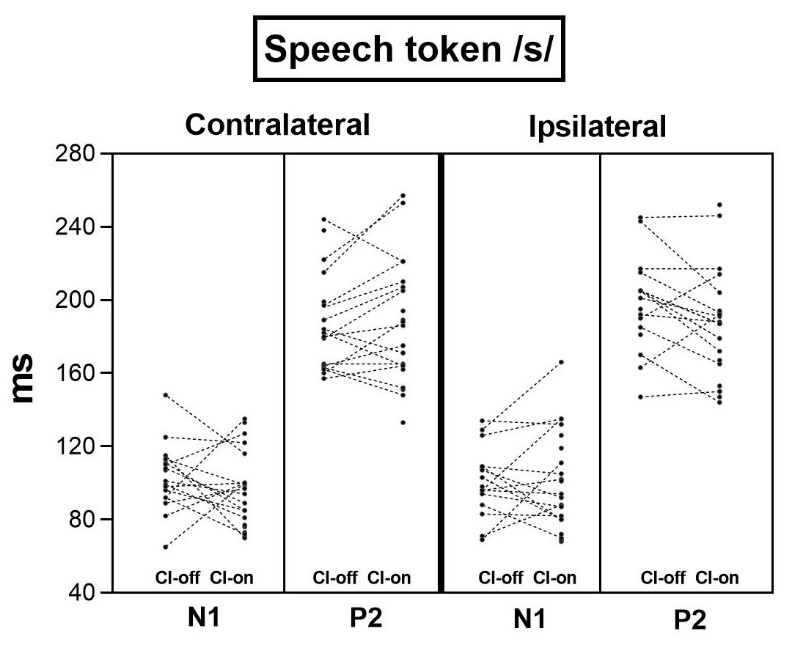

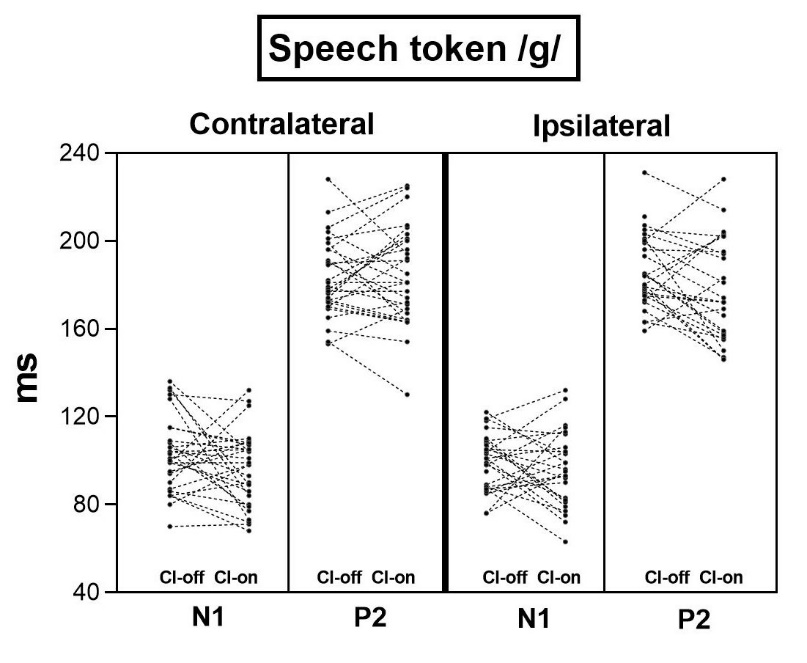

Supplement: S1 Fig — (DOCX) [file pone.0227371.s001.docx]
